# Quasi-linear score for capturing heterogeneous structure in biomarkers

**DOI:** 10.1186/s12859-017-1721-x

**Published:** 2017-06-19

**Authors:** Katsuhiro Omae, Osamu Komori, Shinto Eguchi

**Affiliations:** 1 Department of Statistical Science, The Graduate University for Advanced Studies, 10-3, Midoricho, Tachikawa, Tokyo, 190-8562 Japan; 2 Department of Electrical, Electronic and Computer Engineering, University of Fukui, Fukui, Japan; 3 The Institute of Statistical Mathematics, Tokyo, Japan

**Keywords:** Discriminant analysis, Heterogeneity, Kolmogorov-Nagumo average, Prediction

## Abstract

**Background:**

Linear scores are widely used to predict dichotomous outcomes in biomedical studies because of their learnability and understandability. Such approaches, however, cannot be used to elucidate biodiversity when there is heterogeneous structure in target population.

**Results:**

Our study was focused on describing intrinsic heterogeneity in predictions. Because heterogeneity can be captured by a clustering method, integrating different information from different clusters should yield better predictions. Accordingly, we developed a quasi-linear score, which effectively combines the linear scores of clustered markers. We extended the linear score to the quasi-linear score by a generalized average form, the Kolmogorov-Nagumo average. We observed that two shrinkage methods worked well: ridge shrinkage for estimating the quasi-linear score, and lasso shrinkage for selecting markers within each cluster. Simulation studies and applications to real data show that the proposed method has good predictive performance compared with existing methods.

**Conclusions:**

Heterogeneous structure is captured by a clustering method. Quasi-linear scores combine such heterogeneity and have a better predictive ability compared with linear scores.

**Electronic supplementary material:**

The online version of this article (doi:10.1186/s12859-017-1721-x) contains supplementary material, which is available to authorized users.

## Background

In recent years, biomedical data have become complicated and high-dimensional [1, 2]. For example, a single human gene expression dataset contains tens of thousands of features, many of which are highly correlated [3]. In addition, large mixed datasets are crucial for personalized treatment, in which the optimal treatment strategy is determined based on a dataset that combines a very large number of prognostic factors [4].

From the viewpoint of statistical machine learning, supervised and unsupervised learning methods play central roles in such biomedical studies [5]. In fact, shrinkage methods such as ridge and lasso are frequently used in the context of prediction [6], and clustering methods are used in the context of interpretation [7], potentially revealing novel findings.

Supervised learning methods are often used to estimate risk scores when predicting dichotomous outcomes. Linear scores are among the most widely used forms because it is easy to learn the predictive score from a training dataset. Moreover, it is easy to understand the estimated score. Linear scores are often evaluated by linear discriminant or logistic regression analysis, and achieve not bad discriminative performance. For example, the study of [8] used a linear score, and their discoveries led to the development of Mammaprint, a diagnostic kit for breast cancer metastasis.

Unsupervised learning methods can also yield beneficial insights in high-dimensional data analysis. For example, [9] used biclustering to reveal more detailed subtypes in breast carcinomas with distinctive gene expression profiles from a group that was previously regarded as a homogenous unit. Their study revealed that in order to understand biodiversity, the heterogeneous structure of the targeted population must be considered, and that such heterogeneity can be clarified by the clustering method. Previously published reviews have described both clustering methods [10] and biclustering algorithms [11].

Several studies have combined supervised and unsupervised learning methods. For example, [12] used clustering to discover different patterns of gene expression in different subgroups. They then derived the respective scores for these groups and achieved good specificity without loss of sensitivity relative to existing diagnostic rules. Sample heterogeneity may result in marker heterogeneity. As a result, different samples in different subgroups may have different intrinsic characteristics in their environmental and genetic factors as demonstrated by the motivated example in the “Methods” section. Such heterogeneity may have unexpected effects on a therapy or treatment which is considered as best practice, and lead to an unfavorable risk in one part of the population. Bravo et al. [13] focused on the marker heterogeneity by detecting the genes that showed different variation between healthy and disease samples. They then defined an *anti-profile* score as the number of hyper-variable genes. Thus, more and more studies have considered heterogeneous structure and reflected this heterogeneity in their predictions. However, the risk scores highlighted by published papers are linear, and heterogeneity is therefore not directly reflected in the score form. In this study, we focused on heterogeneity and determined how to directly reflect this intrinsic characteristic in the score form. We developed the quasi-linear score as a result, which combines linear scores as a Kolmogorov-Nagumo average [14, 15], enabling us to reflect the clustering result naturally, because it is based on separated feature vectors.

The rest of this paper is organized as follows. In the “Methods” section, we first present a motivated example of gene expression data and develop the quasi-linear score. Heterogeneity is observed via the clustering method, and we define the quasi-linear score to reflect gene clusters with a generalized average form. We also formulate the quasi-linear logistic model and discuss the difference between the linear and quasi-linear scores. We subsequently evaluated our method by numerical simulations and applications to real datasets. We refer to the relationship between the quasi-linear score and traditional combined approaches in “Discussion” section. All technical details given as Appendix are available in Additional file 1.

## Methods

### Motivation and derivation

**Fig. 1:**
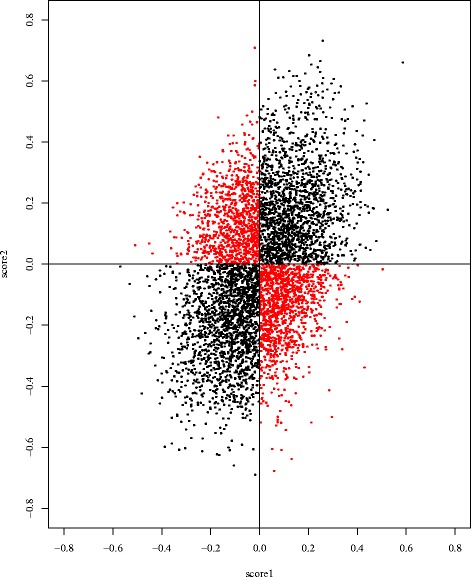
t-statistic values for two datasets from van’t Veer et al. (2002). The *red points* show the genes with sign mismatched t-values for these data

**Fig. 2:**
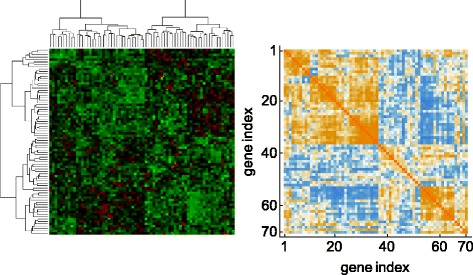
The hierarchical clustering and the correlation matrix of 70 genes for the dataset from van’t Veer et al. (2002). The figure shows the clustering result (*upper*) and the correlation matrix (*lower*). There are 70 rows representing genes and 78 columns representing samples (*upper*) and the gene expression data ranging from *green* (negative) to *red* (positive) are displayed. Elements of the correlation matrix (*lower*) ranging from *blue* (negative) to *yellow* (positive) are displayed

We studied the gene expression dataset from [8]. This dataset is derived from 51 non-metastatic and 46 metastatic breast cancer patients. In their study, the linear score was evaluated to discriminate metastatic events. Because estimation of the predictive linear score is often achieved by a diagonal Fisher’s linear discriminant analysis (DLDA) [16], we considered applying DLDA to this dataset. Because the coefficients of the linear score estimated by DLDA correspond to the t-statistic values, we checked the t-statistics directly for the purpose of visualization. If the data have heterogeneous structure, it can be clarified by observing the difference between two divided, independent datasets. Therefore, we divided the full data into two independent sets, data1 and data2, before calculating the t-statistics for each of them separately. Figure 1 shows the correspondence of the t-statistics. Some genes had no consistency in the signs of their t-values, indicating that some samples from the metastatic group had higher expression, whereas other samples had lower expression, relative to the non-metastatic group. This phenomenon may be caused by heterogeneous factors [17]. In fact, due to the existence of multiple subtypes of breast cancer, this disease is known to exhibit heterogeneity [9]. For such heterogeneous data, clustering methods should work well, as shown in [9]. We applied clustering according to the Ward’s method [18], as shown in Fig. 2, which highlights the results of clustering and the correlation matrix arranged by the clustered genes. Although biclustering result was not suggestive of the heterogeneity in appearance, it was observed via the correlation matrix. Some genes are strongly correlated with others in the same cluster. Thus, we observed the existence of heterogeneity using a t-statistics plot and trends in the expression patterns by clustering. Next, we developed an appropriate score form for discriminating such heterogeneous data based on clustering.

We assume to know decomposition of *p* biomarkers into *K* groups by clustering. Based on these *K* sets of clustered markers, we define a quasi-linear score as

1 $$\begin{array}{@{}rcl@{}} Q=\text{log}\left(\sum_{k=1}^{K} \text{exp}(L_{k})\right), \end{array} $$

where $L_{k}=\alpha _{k}+\beta _{k}^{\top } X_{(k)}$ with the parameters *α*_*k*_,*β*_*k*_, and the marker vector *X*_(*k*)_ for the *k*-th cluster of *k*=1,2,⋯,*K*. When *K*=1, the quasi-linear score *Q* is reduced to the linear score,

2 $$\begin{array}{@{}rcl@{}} L=\alpha+\beta^{\top} X, \end{array} $$

where *α* and $\beta =\left (\beta _{1}^{\top },\cdots,\beta _{K}^{\top }\right)^{\top }$ are parameters, and *X* is the full vector of *X*_(1)_,⋯,*X*_(*K*)_. We note that the intercepts *α*_*k*_’s in (1) are reduced to the single intercept *α* in (2). Additional file 1: Appendix A gives another parameterization for *Q* in which the intercepts *α*_*k*_ are uniquely decomposed to the overall intercept and weights of the *K* clusters.

When determining how to reflect cluster information in the score form, we had two main considerations: which scores should be integrated, and how integration should be performed. All the linear scores *L*_*k*_ are integrated in the quasi-linear score *Q*. We believe that this is reasonable because there are similar markers in each cluster, and we expect that heterogeneity would be caused by different mixed homogeneous features that are sufficiently described by the linear form. Although such an idea of combining the linear scores has already been proposed by [19] as a composite link, it is different from the quasi-linear score in several ways. One of the most significant differences between them is that the quasi-linear score is defined by disjointed sets of markers. This results in a small number of parameters for the predictive score: the parsimonious expression. The difference in these forms is mentioned in the Discussion. Moreover, the quasi-linear score *Q* summarizes *L*_*k*_ approximating the maximum function. In fact, (1) is equal to a soft maximum function discussed by [20], which can be approximated with

3 $$\begin{array}{@{}rcl@{}} M=\max_{1 \leq k \leq K}L_{k}.  \end{array} $$

Therefore, the quasi-linear score *Q* respects the maximum of *K* linear scores from all clusters. See [21] for a discussion of Eq. (3) as maxout in neural networks.

The relationships among *Q*, *L*, and *M* are clearly evaluated when a tuning parameter, *τ*, is introduced in the quasi-linear score *Q* as

4 $$\begin{array}{@{}rcl@{}} Q_{\tau}=\frac{1}{\tau}\text{log}\left(\sum_{k=1}^{K} \text{exp}(\tau L_{k})\right), \end{array} $$

where *τ* is a positive parameter. If *L*_*k*_ is fixed for all *k*, then the form of (4) is defined solely by the tuning parameter *τ*. When *τ* is equal to 1, *Q*_*τ*_ is equal to the quasi-linear score *Q* by definition. When *τ* goes to infinity, *Q*_*τ*_ is simply the maximum score *M*. When *τ* goes to 0, *Q*_*τ*_ is equivalent to the linear score *L*. Thus, these are unified by the hardness of approximation to the maximum function. More details are provided in Additional file 1: Appendix B. The characteristics of the quasi-linear score *Q* are understood by a more general expression of (1) : $G=\phi ^{-1} (\sum _{k=1}^{K} \phi (L_{k}))$, where *ϕ* is an invertible function. We define *G* as a generalized quasi-linear score because the form is the generalized mean called the Kolmogorov-Nagumo average. If we take a simple average, *ϕ*(*z*)=*z*, then the generalized quasi-linear score *G* corresponds to the linear score *L*. In this sense, the linear score *L* is a simple mean, and the quasi-linear score *Q* is a generalized mean of linear scores *L*_*k*_ averaged by the exponential function. Although the simplest integration of clustered information is achieved by a simple average, resulting in the linear score form, it is intuitively unsatisfying because the predictive performance of these linear scores *L*_*k*_ differs among the clusters. A cluster that strongly discriminates the outcome on its own should be respected in comparison with the other clusters. If only the cluster with the highest linear score is reflected in the prediction, it is described by the maximum score *M*. However, this situation is still not ideal, because only one cluster is reflected in the prediction, and form (3) is difficult to handle mathematically because it is not differentiable when two or more linear scores are equal. Consequently, parameter estimation becomes impossible. The quasi-linear score *Q* is therefore naturally derived, and it is reasonable for discriminant analysis of the heterogeneous data because the quasi-linear score *Q* plays an important role in cluster selection, as discussed later.

In the following sections, we let *ϕ*(*z*)=exp(*z*), both because this form is approximated by the maximum function, and because it is optimal in the sense of Bayes risk consistency when we consider the simple case in which the label conditional random variables follow a mixture of normal distribution and a normal distribution with equal variance, respectively. Additional file 1: Appendix C provides more detail about the Bayes risk consistency of the situation. Moreover, the exponential function gives us an understandable interpretation of the parameter estimation.

Because we modify only the scoring form, the quasi-linear score *Q* can be applied to all traditional settings in biostatistics, as the generalized linear model with the L_1_ and L_2_ shrinkage methods. In particular, when we combine the quasi-linear score *Q* with lasso shrinkage, the important clusters and variables in each cluster are determined simultaneously because of soft maximum property and L_1_ sparseness. This property provides good performance for the discriminant problem when the data have a much larger number of correlated markers than the number of samples. Therefore, we derive the L_1_ and L_2_ shrinkage quasi-linear logistic model and display the performance of the quasi-linear score *Q* when it is applied to gene expression data.

#### Likelihood for logistic model and maximum likelihood estimation

Consider the data {(*X*_*i*_,*Y*_*i*_);*i*=1,⋯,*n*}, where *X*_*i*_ is a covariate vector and *Y*_*i*_ is a dichotomous outcome which takes 0 or 1 with the *i*-th individual. Assume that we know the decomposition of *X*_*i*_ as *X*_*i*(1)_,⋯,*X*_*i*(*K*)_ with a fixed cluster size *K*, and that this is identical among individuals. We denote the size of *X*_*i*(*k*)_ as *p*_*k*_, where $\sum _{k=1}^{K} p_{k}=p$. We note that the decomposition is given a priori by one of clustering methods for {*X*_*i*_;*i*=1,⋯,*n*}.

We derive the likelihood for a logistic model of the quasi-linear score because of versatility. Therefore, we assume that *Y*_*i*_ is independently distributed according to the Bernoulli distribution with a parameter *π*_*i*_, and consider the logistic model. Below, we denote the quasi-linear score *Q* based on *X*_*i*(1)_,⋯,*X*_*i*(*K*)_ as *Q*_*i*_ for simplicity. The association between *π*_*i*_ and the quasi-linear score *Q*_*i*_ is described by

5 $$\begin{array}{@{}rcl@{}} \text{log}\frac{\pi_{i}}{1-\pi_{i}}=Q_{i}. \end{array} $$

In this setting, the unknown parameters are {*α*_*k*_,*β*_*k*_;*k*=1,⋯,*K*} which specify *Q*_*i*_’s over individuals as in Eq. (1). The log-likelihood function of parameter $\theta =\left (\alpha _{1},\cdots,\alpha _{K}, \beta _{1}^{\top }, \cdots, \beta _{K}^{\top }\right)^{\top }$ is

6 $$\begin{array}{@{}rcl@{}} l(\theta)&=&\sum_{i=1}^{n} Y_{i} Q_{i}-\text{log}(1+\text{exp}(Q_{i})). \end{array} $$

The maximum likelihood estimator (MLE) of *θ* is therefore the solution of

7 $$\begin{array}{@{}rcl@{}} \frac{\partial l(\theta)}{\partial\theta}=W^{\top}(Y-\Pi), \end{array} $$

where *W*=(*∂* *Q*_1_/*∂* *θ*,⋯,*∂* *Q*_*n*_/*∂* *θ*)^⊤^, *Y*=(*Y*_1_,⋯,*Y*_*n*_)^⊤^ and *Π*=(*π*_1_,⋯,*π*_*n*_)^⊤^. The solution is calculated by updating some initial value repeatedly by Fisher’s scoring method as

8 $$ \theta^{(t+1)}=\theta^{(t)}+\left(W^{(t)\top} V^{(t)} W^{(t)} \right)^{-1} W^{(t)\top}\left(Y-\Pi^{(t)}\right),   $$

where $W^{(t)}=\left (\partial Q_{1} / \partial \theta,\cdots,\partial Q_{n} / \partial \theta \right)^{\top } |_{\theta =\theta ^{(t)}}$, $V^{(t)}=\text {diag}\left \{\pi _{1}^{(t)}\left (1-\pi _{1}^{(t)}\right),\cdots,\pi _{n}^{(t)}\left (1-\pi _{n}^{(t)}\right) \right \}$ and $\Pi ^{(t)}=\left (\pi _{1}^{(t)},\cdots,\pi _{n}^{(t)}\right)^{\top }$. The R source code of the parameter estimation of the quasi-linear logistic model is available in Additional file 2. In the framework of a generalized linear model, *Z* ^(*t*)^=*W* ^(*t*)^ *θ* ^(*t*)^+*V* ^(*t*)^ ^−1^(*Y*−*Π* ^(*t*)^) is called the working response, and this algorithm is referred to as the iteratively reweighted least-square method [22] because the Eq. (8) is written as *θ* ^(*t*+1)^=(*W* ^(*t*)⊤^ *V* ^(*t*)^ *W* ^(*t*)^)^−1^ *W* ^(*t*)⊤^ *V* ^(*t*)^ *Z* ^(*t*)^. Thus, the parameter estimation strategy is very similar to the linear-logistic model. However, the estimation is not stable in a high dimensional setting. In such situation, *W* ^(*t*)⊤^ *V* ^(*t*)^ *W* ^(*t*)^ becomes a singular matrix. It is thus difficult to compute the inverse matrix in Eq. (8) for each step. We can avoid the problem by regularization method, just as for the penalized linear logistic model [23, 24].

### L_1_ and L_2_ regularization of the quasi-linear logistic model

The L_2_ penalized log-likelihood is described by

9 $$ l^{\text{ridge}}(\theta,\lambda) = l(\theta)-\frac{1}{2}\lambda_{0} \sum_{k=1}^{K} \alpha_{k}^{2}-\frac{1}{2}\sum_{k=1}^{K} \lambda_{k} \beta_{k}^{\top} \beta_{k}.  $$

We note that we regularized *α*_*k*_’s by *λ*_0_ to avoid computational difficulty in calculating the inverse matrix, although the intercept parameters should not be regularized in the linear logistic model.

A MLE with the ridge regularization of *θ* is calculated by Fisher’s scoring method as

10 $$\begin{array}{@{}rcl@{}} \theta^{(t+1)}&=& \left(W^{(t)\top} V^{(t)} W^{(t)}+R \right)^{-1} W^{(t)\top} V^{(t)}\\ &&\times\left\{W^{(t)\top}\theta^{(t)}+{V^{(t)}}^{-1}\left(Y-\Pi^{(t)}\right)\right\}. \end{array} $$

Here $R=\text {diag}(\lambda _{0} I_{K},\lambda _{1} I_{p_{1}},\cdots,\lambda _{K} I_{p_{K}})\phantom {\dot {i}\!}$, where *I*_*m*_ denotes the identity matrix with size *m*. The derivation of the algorithm is described in Additional file 1: Appendix D in greater detail.

Next we consider L_1_ regularization for the quasi-linear logistic model. The L_1_ penalized log-likelihood is given by

11 $$\begin{array}{@{}rcl@{}} l^{\text{lasso}}(\theta,\lambda) &=& l(\theta)-\sum_{k=1}^{K} \lambda_{k} |\beta_{k}|. \end{array} $$

This form is compatible with the group lasso [25]. We note that the group lasso has a very similar concept in that regularizations are performed for each cluster. However, the score forms are different between the two regularization methods. The comparison of group lasso and quasi-linear score are performed in the “Application” subsection of the “Results”. For the quasi-linear score *Q*, it is computationally difficult to solve the problem of maximization with (11) by a method that involves the inverse matrix. Therefore, we applied the gradient ascent method of [26] by using the directional derivative, which is a simple gradient ascent algorithm based on the components of a score function:

12 $$ \theta^{(t+1)}=\theta^{(t)}+\text{min} \left\{t_{\text{opt}}\left(\theta^{(t)}\right), t_{\text{edge}}\left(\theta^{(t)}\right)\right\}g\left(\theta^{(t)}\right),  $$

where *g*(*θ*)=(*g*_1_(*θ*),⋯,*g*_*p*+*K*_(*θ*))^⊤^,

$$\begin{aligned} t_{\text{edge}}(\theta)=\min_{1+K \leq j \leq p+K}\left(-\frac{\theta_{j}}{g_{j} (\theta)} : \text{sign}(\theta_{j})=-\text{sign}(g_{j}(\theta))\neq 0\right) \end{aligned} $$

and

$$t_{\text{opt}}(\theta)=\frac{|g(\theta)|}{g(\theta)^{\top} \frac{\partial^{2} l(\theta)}{\partial \theta \partial \theta^{\top}}g(\theta)}. $$

Here *g*_*j*_(*θ*)=*l*_*j*_(*θ*) for *j*=1,⋯,*K* and

$$\begin{array}{@{}rcl@{}} g_{j}(\theta)= \left\{\begin{array}{ll} l_{j}(\theta)-\lambda_{k} \text{sign}(\theta_{j}) & \text{if}\ \theta_{j} \neq 0 \\ l_{j}(\theta)-\lambda_{k} \text{sign}(l_{j}(\theta)) & \text{if}\ \theta_{j}=0 \ \ \text{and}\ |l_{j}(\theta)|>\lambda_{k} \\ 0 & \text{otherwise} \end{array}\right. \end{array} $$

for *j*=*K*+1,⋯,*p*+*K*, where sign(*z*) is a sign function, *l*_*j*_ is the *j*-th component of Eq. (7) and *k* denotes the cluster number the *j*-th marker belongs to. In each step, the *t*_opt_ provides the optimal solution of the gradient descent algorithm and *t*_edge_ controls the direction of the gradient so as to avoid changing the signs of the parameters. The vector of the tuning parameters (*λ*_1_,⋯,*λ*_*K*_)^⊤^ is determined by a cross-validation method from candidate sets of parameters.

### Non-linearity of the quasi-linear score

**Fig. 3:**
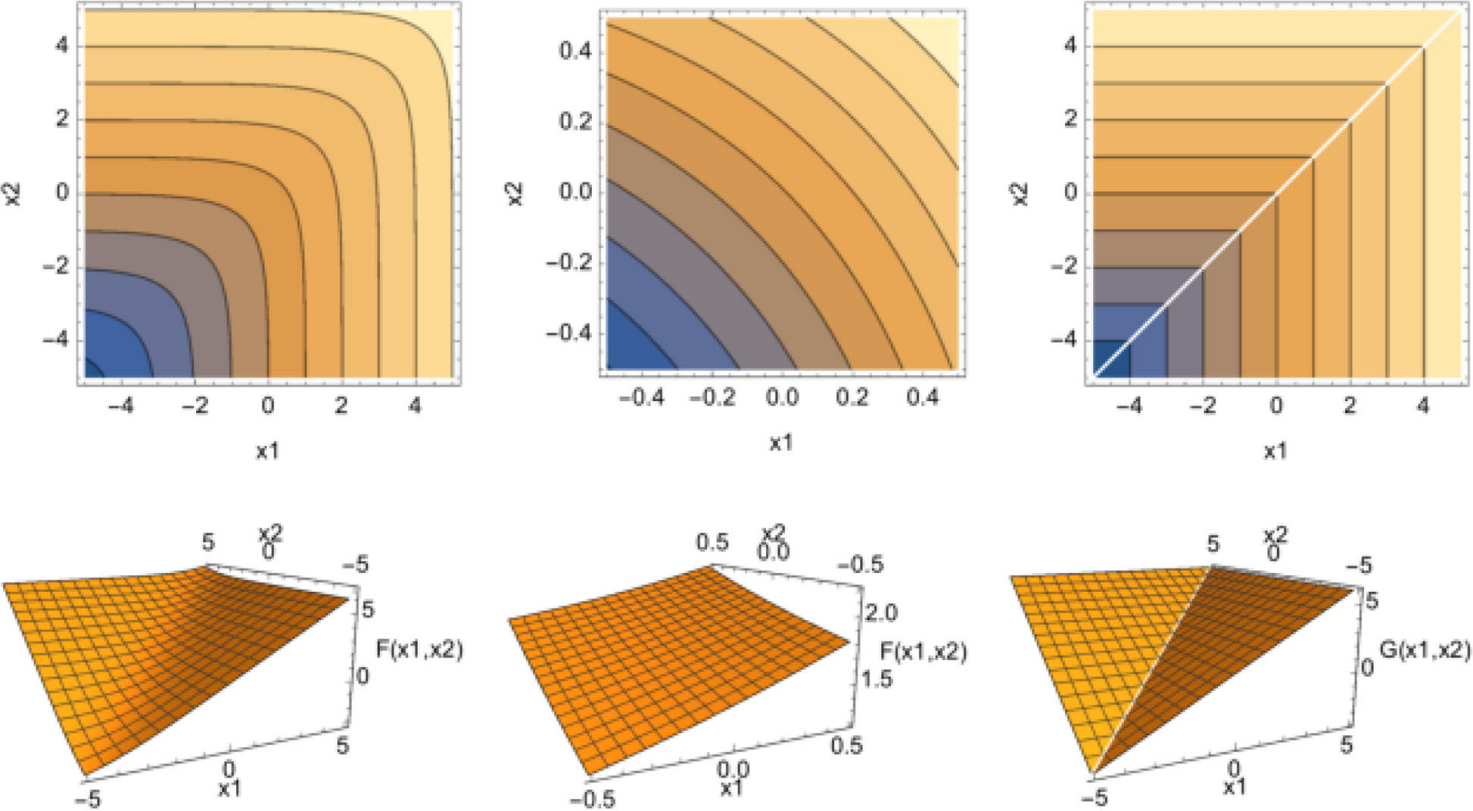
The boundary (*upper*) and contour (*lower*) of the quasi-linear score. *Left* and *center*; *F*(*x*_1_,*x*_2_)=log(exp(1+*x*_1_)+exp(1+*x*_2_)), the *right*; *G*(*x*_1_,*x*_2_)=max(1+*x*_1_,1+*x*_2_). The *center panel* is an expansion around the origin (0,0) of the *left panel*

The quasi-linear score *Q* is non-linear by definition. The non-linearity of the quasi-linear score *Q* can be demonstrated by a simple illustration. Figure 3 shows the fitted curve of *Q* when *p*=*k*=2. In this figure, it looks as if two linear planes, specialized to each sub-space, are connected smoothly. In this case, the linear surface is curved while still maintaining local linearity, thus forming a quasi-linear surface. As an extreme case, let there be only one cluster with strong markers. When all scores are integrated, the information from this cluster should not be affected by the others. The quasi-linear score *Q* makes up this nature because this approximates the maximum function. If there is an *ℓ*,1≤*ℓ*≤*K* such that

13 $$\begin{array}{@{}rcl@{}} L_{\ell} \gg L_{k} \end{array} $$

for *k*≠*ℓ*, then $\sum _{k=1}^{K} \exp (L_{k})\approx \exp (L_{\ell })$, so that *Q* almost equals *L*_*ℓ*_ and the score is almost evaluated by the *ℓ*-th cluster. In such a case, the quasi-linear score *Q* achieves the cluster selection. In the numerical sense, even if the inequality (13) is not very evident, selection is considered to be achieved because the exponential function inflates the input sufficiently. For example, log{exp(5)+ exp(2)+ exp(−1)+ exp(−4)}=5.051, which essentially means that only the first term is reflected in the construction of the quasi-linear score *Q*. Accordingly, *Q*≈*L*_*ℓ*_ if *X* is in a set {*X*:*L*_*ℓ*_=*M*}, say *C*_*ℓ*_. We note that *C*_*ℓ*_ is expressed by the intersection of *K*−1 half planes, such that *C*_*ℓ*_ is a convex polyhedron. Thus the quasi-linear score *Q* is locally linear over disjointed and exhaustive regions of the space of all biomarkers : $\bigcup _{\ell =1}^{K} C_{\ell }$. Thus we observe that the quasi-linear score *Q* is approximately equal to the linear score *L* that dominates over the other *K*−1 scores. This property contrasts with the ordinary linear score, which is the sum of *K* linear scores. In particular, the quasi-linear score *Q* is advantageous in cases where there are predominant sets of separate biomarkers within the space of all biomarkers.

Also, for both logistic models in the parameter estimation steps, we can see the difference between the linear and quasi-linear models reflected in the derivative term as:

14 $$\begin{array}{@{}rcl@{}} \frac{\partial l^{L}\left(\theta^{L}\right)}{\partial \theta^{L}}&=&\left(1, X^{\top}\right)^{\top}, \end{array} $$

15 $$\begin{array}{@{}rcl@{}} \frac{\partial l(\theta)}{\partial \theta}&=&\left(S_{1},\cdots,S_{K},S_{1} X_{(1)}^{\top},\cdots,S_{K} X_{(K)}^{\top}\right)^{\top}, \end{array} $$

where *l* ^*L*^(*θ* ^*L*^) is a log-likelihood function of the linear logistic model with parameter *θ* ^*L*^=(*α*,*β* ^⊤^)^⊤^ and $S_{k}=\text {exp}(L_{k})/\sum _{k=1}^{K} \text {exp}(L_{k})$. A derivation of Eq. (15) is given in Additional file 1: Appendix E. Thus, the data space is decomposed by updated *S*_*k*_ and composed as one unit in each learning step. This concept used in probabilistic models is referred to as the divide and conquer strategy, which is employed in many machine-learning studies as a mixture of expert models [27].

## Results

### Simulation study

We examined the efficiency of the quasi-linear score *Q* using logistic models (QL), compared with the linear score *L* using logistic model (LL). We conducted simulations with five different settings. For each dataset, the samples were divided between the disease group (*Y*=1) and normal group (*Y*=0).

First, to show the consistency of the quasi-linear logistic model without regularization, we used a simple setting that has an optimal solution of the quasi-linear form. In this example, we simulated 1000 random datasets. Each dataset was either small, containing 400 samples, or large, containing 1600 samples. Next, we estimated the parameters using Eq. (8) and checked the consistency.

Second, we examined four high dimensional settings focusing on marker’s selection. The divided populations were considered to have homogeneous or heterogeneous structure, which were described by normal or mixed normal distribution. In these examples, we simulated 1000 random datasets, each containing either 400 or 200 samples for training and test datasets, respectively. For these settings, we use the L_1_ and L_2_ shrinkage method in order to avoid overfitting and hard computation. Below, we define $\boldsymbol {r}_{p}=(r,r,\cdots,r) \in \mathbb {R}^{p}$ for the simple notations.

#### Consistency

In this example, we assumed normality for the normal group and mixture normality for the disease group.

16 $$\begin{array}{@{}rcl@{}} X|(Y=0) &\sim& \mathrm{N}\left(\boldsymbol{0}_{2}^{\top}, I_{2}\right), \quad X|(Y=1)\\ &\sim& \sum\limits_{g=1}^{2} \tau_{g} \mathrm{N}\left(\mu_{1g}, I_{2}\right),\quad\sum\limits_{g=1}^{2} \tau_{g} =1. \end{array} $$

**Fig. 4:**
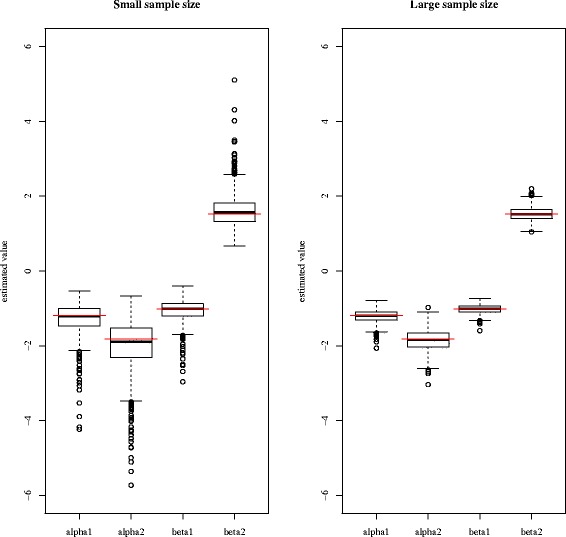
Box plot of the estimated parameters in the simple simulation. *Left*: small sample size setting (*n*=400); *right*: large sample size setting (*n*=1600). The *red lines* show the optimal parameters derived from the true likelihood

We let *μ*_11_=(−1,0)^⊤^ and *μ*_12_=(0,1.5)^⊤^. In this setting, the Bayes optimal form is log(exp(*α*_1_+*β*_1_ *X*_1_)+exp(*α*_2_+*β*_2_ *X*_2_)). Figure 4 shows box plots of estimated parameters for 1000 trials. The optimal parameter derived from the true likelihood is (*α*_1_,*α*_2_,*β*_1_,*β*_2_)=(−1.19,−1.82,−1.00,1.50). The means of the estimated parameters from 1000 trials are (*α*_1_,*α*_2_,*β*_1_,*β*_2_)=(−1.28,−1.99,−1.07,1.61) for the small datasets and (*α*_1_,*α*_2_,*β*_1_,*β*_2_)=(−1.21,−1.85,−1.01,1.53) for the large datasets. We observed that parameter estimation was more precise when the sample size was large, and that the estimated parameters were consistent.

#### High dimensional settings

- (*a*): homo-homo
  In this example we assumed normality for both groups.
  17 $$\begin{array}{@{}rcl@{}} X|(Y=y) \sim \mathrm{N}\left(\mu_{y}, I_{p}\right) \quad (y=0,1). \end{array} $$
  We had three settings: (1) $p=2,\mu _{0} =\boldsymbol {0}_{2}^{\top },\mu _{1}=\boldsymbol {1}_{2}^{\top }$, (2) $p=100, \mu _{0} = \boldsymbol {0}_{100}^{\top },\mu _{1}=\boldsymbol {0.1}_{100}^{\top }$, (3) $p=100, \mu _{0} = \boldsymbol {0}_{100}^{\top },\mu _{1}= \boldsymbol {0.5}_{100}^{\top }$. For the quasi linear score *Q*, we assumed the misspecification of heterogeneous structure, as *K*=2 and *p*_1_=*p*_2_=1 for (1) or *p*_1_=*p*_2_=50 for (2) and (3).

- (*b*): homo-hetero
  In this example, we assumed normality for the normal group and mixed normality for the disease group.
  18 $$\begin{array}{@{}rcl@{}} X|(Y=0) &\sim& \mathrm{N}(\mu_{0}, I_{p}), \quad X|(Y=1)\\ &\sim& \sum_{g=1}^{G} \tau_{g} \mathrm{N}(\mu_{1g}, I_{p}), \quad \sum_{g=1}^{G} \tau_{g} =1. \end{array} $$
  We had four settings. In (1) and (2), we let *G*=2, *p*=100, *τ*_1_=*τ*_2_=0.5, $\mu _{0}=\boldsymbol {0}_{100}^{\top }$. In (3) and (4), we let *G*=3, *p*=100, *τ*_1_=*τ*_2_=*τ*_3_=1/3, $\mu _{0}=\boldsymbol {0}_{100}^{\top }$. The mean parameter for the disease group was set as (1) *μ*_11_=(−1,***0***_99_)^⊤^, *μ*_12_=(***0***_50_,1.5,***0***_49_)^⊤^, (2) *μ*_11_=(***−1***_10_,***0***_90_)^⊤^, *μ*_12_=(***0***_50_,***1.5***_10_,***0***_40_)^⊤^, (3) *μ*_11_=(−1.5,***0***_99_)^⊤^, (4) *μ*_11_=(−***1.5***_3_,***0***_97_)^⊤^, *μ*_12_=(***0***_34_,***1.5***_3_,***0***_63_)^⊤^, *μ*_13_=(***0***_67_,***1***_3_,***0***_30_)^⊤^. For the quasi-linear score *Q* we assumed the correct specification of heterogeneous structure as *K*=*G* and *p*_1_=*p*_2_=50 or *p*_1_=34,*p*_2_=*p*_3_=33.

- (*c*): hetero-hetero
  In this example, we assumed mixed normality for both groups.
  19 $$\begin{array}{@{}rcl@{}} X|(Y=y) &\sim& \sum_{g=1}^{G} \tau_{yg} \mathrm{N}(\mu_{yg}, I_{p}),\\ &&\sum_{g=1}^{G} \tau_{yg} =1\ \ (y=0,1). \end{array} $$
  We used the following settings: *G*=2, *p*=100, *τ*_*yg*_=0.5 (*y*=0,1, *g*=1,2), $\mu _{01}=\boldsymbol {0}_{100}^{\top }$, *μ*_02_=(***0***_50_,***0.3***_10_,***0***_40_)^⊤^, *μ*_11_=(***0.5***_50_,***0***_50_)^⊤^, *μ*_12_=(***0***_50_,***0.8***_50_). For the quasi-linear score *Q* we assumed to specify there are heterogeneous structure as *K*=2 and *p*_1_=*p*_2_=50.

- (*d*): correlated
  In this example, we assumed normality for the normal group and mixed normality for the disease group.
  20 $$\begin{array}{@{}rcl@{}} X|(Y=0) &\sim& \mathrm{N}(\mu_{0}, \Sigma), \quad X|(Y=1)\\ &\sim& \sum_{g=1}^{G} \tau_{g} \mathrm{N}(\mu_{1g}, \Sigma), \quad \sum_{g=1}^{G} \tau_{g} =1. \end{array} $$
  The variance assumption was based on a real dataset, as shown in Fig. 2. We used the following settings: (1) $G\,=\,2, p\,=\,70, \tau _{1}=\tau _{2}=0.5, \mu _{0}=\boldsymbol {0}_{70}^{\top }, \mu _{11}=(-\boldsymbol {0.5}_{5},\boldsymbol {0}_{65})^{\top }$, *μ*_12_=(***0***_35_,***1***_5_,***0***_30_)^⊤^, $\Sigma = \left (\begin {array}{cc} \Sigma _{1} & \Sigma _{2} \\ \Sigma _{2}^{\top } & \Sigma _{1} \end {array}\right)$, where *Σ*_1_=0.7*I*_35_+0.3*J*_35_,*Σ*_2_=−0.15*J*_35_, where *J*_*m*_ is a matrix of size *m* of which all components are 1. For the quasi-linear score *Q* we assumed to specify there are heterogeneous structure as *K*=2 and *p*_1_=*p*_2_=50.

Table 1 (*a*) summarizes the AUC value of the test datasets for the (*a*) settings. We note that the linear score *L* is optimal, in terms of the likelihood ratio, under this assumption. However, the quasi-linear score *Q* is not less than the simple linear score *L* regardless of the misspecified structure. This is because the quasi-linear score *Q* includes the local linear boundary, and almost of all data points are fitted to it. As a result, the predictions based on the quasi-linear score *Q* were not so mismatched. Table 1 (*b*) summarizes the AUC values of the test datasets for the (*b*) settings. We note that the quasi-linear score *Q* is Bayes-optimal under this assumption. Unlike in a situation that involves checking for consistency, the quasi-linear score *Q* succeeded in making a difference in performance relative to the ordinary linear score *L*. As the numbers of effective explanatory valuables increased, the difference in predictive performance between the quasi-linear and linear scores also grew. In these settings, the L_1_ shrinkage method performed well, because the number of effective explanatory variables was small compared to the number of noisy variables. Table 1 (*c*) summarizes the AUC value of test datasets for the (*c*) setting. When we assumed normal heterogeneity for both groups, the optimum form of the score was no longer simple, and differs from the linear and the quasi-linear forms. However, the quasi-linear score *Q* also worked well in this setting. This result indicates that the quasi-linear score *Q* should have good predictive performance relative to the linear score *L* in complex heterogeneous settings like real datasets. Table 1 (*d*) summarizes the AUC value of the test datasets for the (*d*) setting. The quasi-linear score *Q* also worked well in this setting.

**Table 1:** Estimated AUC (standard deviation) of 1000 repetitions

			LL		QL		LR
			Ridge	Lasso	Ridge	Lasso	No penalty
(*a*)	Homo-homo	(1)	0.841 (0.027)	0.840 (0.027)	0.818 (0.029)	0.818 (0.029)	0.842 (0.027)
		(2)	0.690 (0.039)	0.665 (0.040)	0.679 (0.041)	0.685 (0.029)	0.760 (0.033)
		(3)	0.999 (0.001)	0.997 (0.002)	0.999 (0.001)	0.999 (0.001)	0.999 (0.001)
(*b*)	Homo-hetero	(1)	0.641 (0.040)	0.675 (0.038)	0.659 (0.039)	0.725 (0.036)	0.754 (0.034)
		(2)	0.953 (0.014)	0.960 (0.013)	0.985 (0.007)	0.963 (0.016)	0.986 (0.006)
		(3)	0.616 (0.040)	0.642 (0.040)	0.634 (0.040)	0.668 (0.040)	0.740 (0.035)
		(4)	0.757 (0.033)	0.796 (0.032)	0.817 (0.029)	0.827 (0.029)	0.890 (0.022)
(*c*)	Hetero-hetero	(1)	0.713 (0.039)	0.697 (0.047)	0.766 (0.035)	0.752 (0.039)	0.824 (0.029)
(*d*)	Correlated	(1)	0.762 (0.034)	0.741 (0.037)	0.781 (0.033)	0.736 (0.055)	0.841 (0.024)

### Application

We applied our method for two datasets, namely breast cancer and prostate cancer data. For both types of datasets, two independent datasets were used as training and testing to evaluate the predictive ability by test AUC. First, we compared the test AUC among decision tree (DT), random forest (RF), support vector machine (SVM), naive Bayes (NB), group lasso (GL), neural network (NN), L_1_ or L_2_ penalized linear logistic (LL1, LL2) and L_1_ or L_2_ penalized quasi-linear logistic (QL1, QL2). Performance was evaluated by the test AUC and the 95% CIs of the test AUC based on 2000 bootstrapping sampling, as described in [28]. The tuning parameters were determined with a grid search and resampling method as needed. Second, the stability for marker selection was compared among LL1, QL1 and GL. We used a similarity index proposed by [29] defined by *S*(*A,B*)=|*A*∩*B*|/|*A*∪*B*|, where *A* and *B* are subsets of marker index set, and |*A*| is a cardinality of the set *A*. *S* takes a value between 0 and 1 whose high value means high stability. We evaluated the stability measure by $\frac {2}{R(R-1)}\sum _{i=1}^{R-1}\sum _{j=i+1}^{R} S(M_{i},M_{j})$, where *M*_1_,⋯,*M*_*R*_ are sets of the selected marker for *R* bootstrap sample sets from the training data set. *R* was set to 100 below.

#### Breast cancer data

The training dataset was taken from [8] and the test datasets were from [30]. Yan et al. [28] used these datasets and compared the AUCs by the linear score *L*, which they evaluated by traditional methods as well as methods they proposed. We focused on the 70 genes detected by [8], as in [28]. These datasets include 78 patients in one and 307 patients in the other. For QL, grouping of 70 genes was based on the Ward’s clustering method only by training dataset. We had two options for dividing all the genes into clusters. In the first option, the 70 genes were divided into two clusters, one with 36 and the other with 34 genes. In the second option, the 70 genes were divided into three clusters of 36, 16, and 18 genes. For GL, We used two clusters option.

**Fig. 5:**
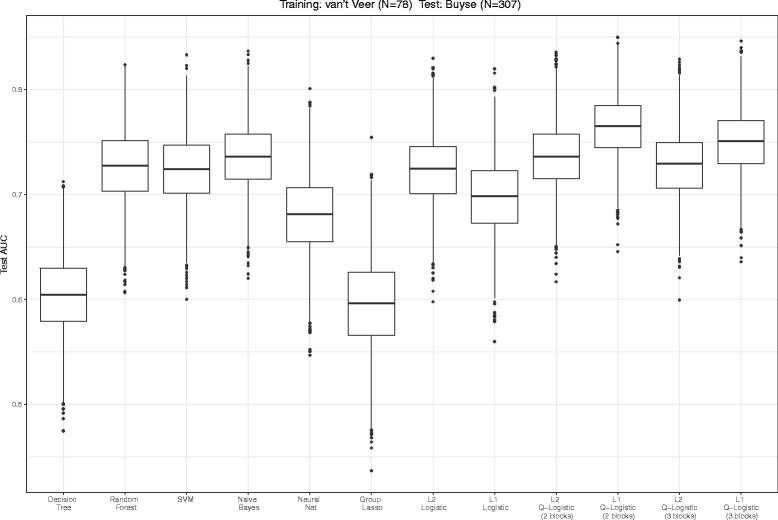
Box plots of the test AUC for all comparative methods by breast cancer data

Figure 5 displays the estimated AUC for the test dataset. QL1 and QL2 performed better than LL1, LL2 and any other non-linear methods, and the highest test AUC was obtained when we used QL1 based on two clusters. The test AUC of the quasi-linear score did not change for different cluster sizes (*K*=2 and *K*=3). The numbers of selected markers in LL1, QL1 (*K*=2), QL1 (*K*=3) and GL were 14, 14, 24 and 70, respectively. Similarly, the stability measures were 0.323, 0.320, 0.399 and 0.960, respectively. The stability did not differ between LL1 and QL1 greatly. We note that GL almost did not shrink any coefficients to zero in this setting.

**Fig. 6:**
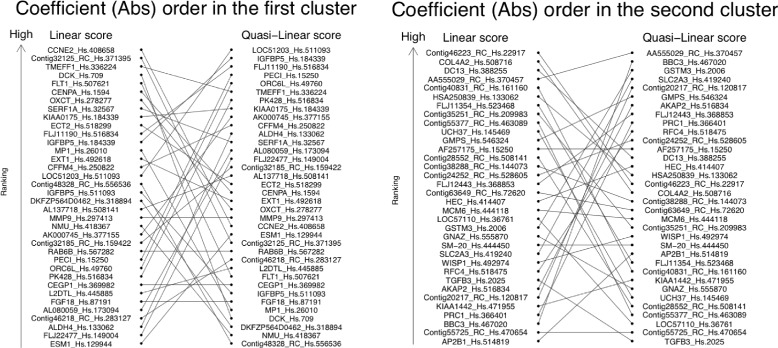
Ranking of the absolute values of the coefficients within the cluster with ridge regularization

When we use the linear score *L*, the absolute value of the coefficients of each marker reflects the order of importance of all markers for prediction. Therefore, the linear score is understandable in the sense that we can recognize strong markers. This is no longer a consideration when we use a generalized non-linear score. However, the quasi-linear score enables us to compare coefficients within the same cluster. An example is shown in Fig. 6, which displays the ranking of the absolute values of the estimated coefficients by the ridge regularization method based on the existence of two clusters. The gene labels are arranged in order of the rankings. We observed that *Q* and *L* gave quite different rankings. This result shows that the quasi-linear score would produce different interpretations for the relationship between the markers.

**Fig. 7:**
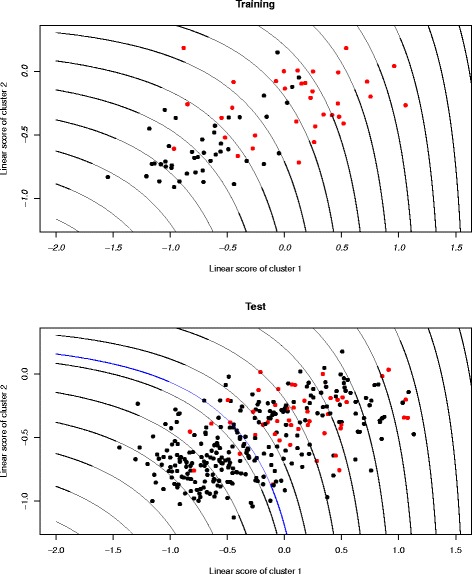
Learning and fitted plot for the training and test dataset when using the quasi-linear score of two clusters with lasso regularization. The horizontal and vertical axis are the linear scores of the first and second cluster. *Red points* indicate the metastatic group and *black points* indicate the control group. *Curve lines* are contours of the quasi-linear score and *blue line* shows cut-off value based on Youden-index

Figure 7 shows learning and fitting of the quasi-linear score *Q* using the lasso regularization method. The score distributions in the training and test datasets were quite well-matched. Figure 7 shows that the quasi-linear score of two clusters with L_1_ regularization will work well if we give a cut-off value for binary decisions. For example, the test error rates of *Q* and *L* were 37.8% and 45.0%, respectively, when we used the Youden-index [31].

Although the quasi-linear score *Q* is approximately equivalent to the maximum function, the two are numerically different. In fact, the test AUC of the quasi-linear score with the lasso regularization method when we assumed two clusters was 0.752, and the corresponding maximum score *M* is 0.745, so that the smooth non-linearity of the quasi-linear form produced good predictive performance

**Fig. 8:**
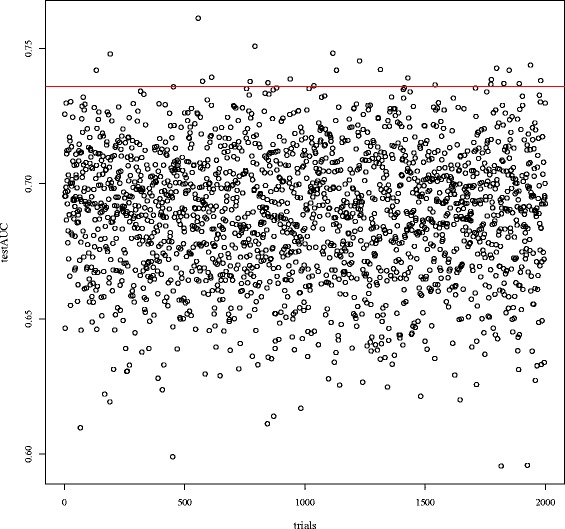
Test AUCs by the quasi-linear score for the dataset from Buyse et al. (2006). The score is learning by randomly divided genes subsets for the dataset from van’t Veer *et al* (2002). The *red line* is the test AUC by the quasi-linear score, which consists of subsets of genes clustered by unsupervised learning

The elastic net shrinkage method [32], which combines the lasso and ridge shrinkage methods, is among the most frequently used. When we combined the quasi-linear score and the elastic net regularization, the number of the tuning parameters was inflated. Although we used the elastic net experimentally for the application for some selected parameters, the predictive performance was not significantly different from the performance obtained with either the lasso or ridge. Detailed results are summarized in Table 2. Moreover, to check the utility of the unsupervised clustering, we randomly divided the 70 genes into two subsets of 36 and 34 genes, and applied QL2 for the test dataset (2000 times). Figure 8 shows that clustered subsets (red line) performs better than randomly divided subsets. Thus, unsupervised clustering naturally benefits supervised learning via the quasi-linear form.

**Table 2:** Estimated AUC (95% confidence interval) by elastic net shrinkage; training dataset from [8], test dataset from [30]

	LL	QL (*K*=2)
*ε*=0.25	0.732 (0.665, 0.796)	0.755 (0.691, 0.814)
*ε*=0.50	0.723 (0.655, 0.788)	0.754 (0.691, 0.813)
*ε*=0.75	0.707 (0.636, 0.776)	0.748 (0.684, 0.807)

A parameter *ε* denotes the proportion of ridge regularization to lasso regularization

#### Prostate cancer data

**Fig. 9:**
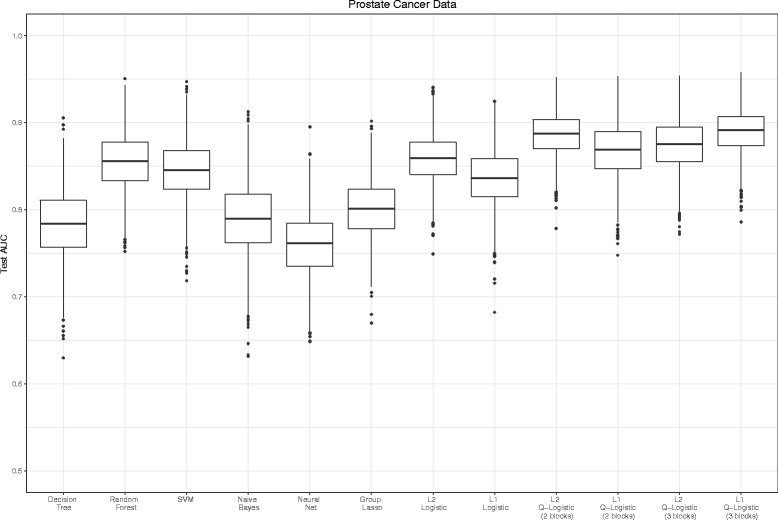
Box plots of the test AUC for all comparative methods by prostate cancer data

The data set was taken from [33] which contains expression data for 6144 genes obtained from 455 prostate cancer tumors. The tumors were from 103 subjects determined to be fusion status-positive and 352 subjects determined to be fusion status-negative. We randomly divided the whole dataset into two independent datasets with the same number of tumor samples (training and test data) while maintaining the ratio of positive to negative statuses. First, we selected 100 relevant genes which had top 100 absolute value of t-statistic between the two statuses using only the training dataset. Such marker preselection has been performed in many studies [34]. For QL, grouping of 100 genes was based on the Ward’s clustering method only by training dataset. We had two options for dividing all the genes into clusters. In the first option, the 100 genes were divided into two clusters, one with 81 and the other with 19 genes. In the second option, the 100 genes were divided into three clusters of 25, 56, and 19 genes. For GL, We used two clusters option. We then compared the test AUC among all comparative methods. Figure 9 displays the estimated AUC for the test dataset. As well as the application for breast cancer data, QL1 and QL2 performed better than any other comparative methods. The numbers of selected markers in LL1, QL1 (*K*=2), QL1 (*K*=3) and GL were 31, 38, 67 and 100, respectively. Similarly, the stability measures were 0.361, 0.993, 0.982 and 1.00, respectively. The stability of QL1 was higher than LL1. We note that GL almost did not shrink any coefficients to zero as application for breast cancer data set.

## Discussion

We focused on heterogeneous structure and determined how to reflect such heterogeneity in the score function defined in (1). For this purpose, the quasi-linear score was derived as the generalized mean called the Kolmogorov-Nagumo average. The quasi-linear form is also called a soft maximum function or log-sum-exp function [35]. In machine learning, the softmax function is often used as a differentiable approximation of the maximum. In computer science, the log-sum-exp function is used to avoid computational problems such as overflow. The non-linearity of the quasi-linear score is explained by the soft maximum function. The quasi-linear score achieves cluster selection because of the soft maximum property as discussed in the subsection of “Non-linearity of the quasi-linear score”. This formulation does not require any prior information or assumption to separate markers into clusters, because this is achieved by the unsupervised learning step.

The quasi-linear score is based on the idea of combining predictors, which is related to several ideas in the literature. For example, a mixture of expert models suggest the idea of decomposing input space [27], in which the model divides the problem space probabilistically and the scores learned in all sub-spaces are combined. The quasi-linear score utilizes the information given by the clustering method to reflect the heterogeneity of markers and combines the linear scores of all clusters. Hence, it relies on the disjointed decomposition of the markers. The method of combining linear scores was also discussed in [19], known as composite links, which assumes that the score is formed by a weighted sum of block-wise markers. Unlike the generalized linear model, the composite link model does not restrict the use of the single link function. In a special case, the composite link logistic model corresponds to the quasi-linear logistic model. However, these ideas differ in that the composite link considers the sum of the linked linear scores whereas the quasi-linear score considers a linkage of the summarization of linear scores in all clusters. The key in our proposal is to model heterogeneity using information from the clustering method, thereby connecting supervised learning with unsupervised learning without any assumption via a change in the score form from the simple average to the Kolmogorov-Nagumo average.

For future work, we intend to extend some fixed settings presented in this report. These include the choice of the clustering method, the size of the markers and clusters, the set of tuning parameters, the type of outcomes, and the format of the targeted data. Because the quasi-linear score can be defined by any decomposition ideas, the performance should be evaluated by clustering methods other than Ward’s method, such as the k-means method [36]. Moreover, we need to investigate the sizes of markers and clusters, and the number of candidate sets of tuning parameters in addition to the parameter *τ* in (4), to obtain a more flexible form of the quasi-linear function. Although we applied and evaluated the proposed method after marker preselection in Application, the performance should be evaluated in much higher dimensional setting. An especially big concern is how to decide the cluster size for the quasi-linear score. As described in the “Application” subsection, the quasi-linear score by cluster size 2 gave the best predictive performance for breast cancer data, and adding more clusters yielded no improvement. Figure 2 supports this result because whole markers were divided into two primary clusters. It is necessary to develop an objective index of definite cluster size selection for general applications.

The quasi-linear score would be also applicable in a case of the continuous outcomes and in a regression model, although we focused on binary outcomes and the logistic model in this study. The performance of the quasi-linear score would be exhibited in the mixed large dataset, which would play an important role in biomedical studies in the near future, because such data must be heterogeneous. Furthermore, our method is not limited to biomedical data, and could also be beneficial for analyzing any data that have heterogeneous structure.

## Conclusions

In this paper, we focused on heterogeneous structure. Such heterogeneity was captured well by a clustering method. The quasi-linear score was naturally derived by Bayes risk consistency between mixed and standard normal distributions. Moreover, the quasi-linear score approximates the maximum function and plays an important role in selecting the most effective cluster for prediction from given clusters. The quasi-linear score has better predictive ability compared to linear score as shown in simulation studies and applications to real data.

## Additional files

Additional file 1 Technical derivations. In this file, we perform some technical derivations and evaluations for quasi-linear score: parameterization; the relationship with linear and maximum score; the Bayes risk consistency; L_1_ and L_2_ regularization methods; the derivatives. (PDF 45 kb)

Additional file 2 R source code of the parameter estimation of the quasi-linear logistic model. In this file, we introduce the R source code of the parameter estimation of the quasi-linear logistic model, which was used for Simulation and Application. (PDF 12 kb)

## Abbreviations

AUC: Area under curve
DLDA: Diagonal Fisher’s linear discriminant analysis
MLE: maximum likelihood estimator
