# Supplementary material for: Quasi-linear score for capturing heterogeneous structure in biomarkers
Source: BMC Bioinformatics. 2017 Jun 19;18:308. doi: 10.1186/s12859-017-1721-x (PMC5477283; doi:10.1186/s12859-017-1721-x)
Supplement: Supplementary file 1 — Technical derivations. In this file, we perform some technical derivations and evaluations for quasi-linear score: parameterization; the relationship with linear and maximum score; the Bayes risk consistency; L1 and L2 regularization methods; the derivatives. (PDF 45 kb) [file 12859_2017_1721_MOESM1_ESM.pdf]

Appendix A : Parameterization of the quasi-linear score

The quasi-linear score Q has parameters α_k's and β_k's in (1). These are interpreted as the intercept and slope vector of the linear score in each cluster. It is written as

$$Q = \alpha^{'} + \log\left(\sum_{k=1}^{K} w_k \exp(\beta_k^{\top} X_k)\right), \tag{A.1}$$

where $\alpha^{'} = \log\left(\sum_{k=1}^{K}\exp(\alpha_k)\right)$ and $w_k = \exp(\alpha_k)/\sum_{k=1}^{K}\exp(\alpha_k)$. We note that $\sum_{k=1}^{K} w_k = 1$. Thus the intercepts α_k's are one-to-one correspondence to the overall intercept and the weights of the K clusters.

Appendix B : The relationship among linear, quasi-linear and maximum score

We provide the relationship among the forms of (1), (2), and (3) via (4), the quasi-linear score in which the tuning parameter τ is introduced. When $\tau = 1$,

$$\begin{aligned} Q_1 &= \log\left(\sum_{k=1}^{K} \pi_k \exp(L_k)\right) \\ &= \log\left(\sum_{k=1}^{K} \exp(L_k^{'})\right), \end{aligned} \tag{B.1}$$

where $L_k^{'} = \alpha_k^{'} + \beta_k^{\top} X_{(k)}$ and $\alpha_k^{'} = \alpha_k + \log(\pi_k)$. Thus, the form of (1) is a parsimonious expression because the intercept parameter includes the proportional weight of each cluster for all clusters.

By the definition of maximum function, it holds obviously following that

$$\max_{1\le i\le n} \exp(z_i) \le \sum_{i=1}^{n} \exp(z_i) \le n \max_{1\le i\le n} \exp(z_i) \tag{B.2}$$

for any real values $z_1, z_2, \cdots, z_n$. Take a log-transformation and we get that

$$\max_{1\le i\le n}(z_i) \le \log\left(\sum_{i=1}^{n} \exp(z_i)\right) \le \max_{1\le i\le n}(z_i) + \log(n). \tag{B.3}$$

Using (B.3), we obtain

$$\max_{1\le k\le K}(\tau L_k^{'}) \le \log\left(\sum_{k=1}^{K} \exp(\tau L_k^{'})\right) \le \max_{1\le k\le K}(\tau L_k^{'}) + \log(K),$$

which can be written as

$$\max_{1\le k\le K}(L_k^{'}) \le \frac{1}{\tau}\log\left(\sum_{k=1}^{K} \exp(\tau L_k^{'})\right) \le \max_{1\le k\le K}(L_k^{'}) + \frac{1}{\tau}\log(K). \tag{B.4}$$

Therefore, when τ goes to infinity, Q_τ converges to M by a sandwich theorem.

When τ goes to 0, we see by l'Hôpital's rule that

$$\begin{aligned}\lim_{\tau\to+0} Q_\tau &= \lim_{\tau\to+0} \frac{\log\left(\sum_{k=1}^{K}\pi_k \exp(\tau L_k)\right)}{\tau} \\ &= \lim_{\tau\to+0} \frac{\sum_{k=1}^{K}\pi_k L_k \exp(\tau L_k)}{\sum_{k=1}^{K}\pi_k \exp(\tau L_k)} \\ &= \sum_{k=1}^{K}\pi_k L_k \\ &= \sum_{k=1}^{K}\pi_k\alpha_k + (\pi_k\beta_k)^\top X_{(k)} \\ &= \tilde{\alpha} + \tilde{\beta}^\top X, \end{aligned} \tag{B.5}$$

where $\tilde{\alpha} = \sum_{k=1}^{K}\pi_k\alpha_k$ and $\tilde{\beta} = (\pi_1\beta_1^\top, \cdots, \pi_K\beta_K^\top)^\top$. The form of (B.5) is equivalent to the linear score. Thus, Q, L and M are unified by Q_τ with the parameter τ, which controls the hardness of approximation for the maximum function.

Appendix C : The Bayes risk consistency

Assume that $X|(Y=1) \sim \sum_{k=1}^{K}\pi_k f(\mu_k, \Sigma)$ and $X|(Y=0) \sim f(\mathbf{0}, \Sigma)$, where $f(\mu, \Sigma)$ denotes a normal density with mean μ and variance matrix Σ. Then, the true log ratio of class posteriors is

$$\begin{aligned}\log\frac{\Pr(Y=1|X)}{\Pr(Y=0|X)} &= \log\frac{p_1\sum_{k=1}^{K}\pi_k f(\mu_k,\Sigma)}{p_0 f(\mathbf{0},\Sigma)} \\ &= \log\left(\sum_{k=1}^{K}\exp\left(\log\pi_k - \frac{1}{2}\mu_k^\top\Sigma^{-1}\mu_k + \log\frac{p_1}{p_0} + (\Sigma^{-1}\mu_k)^\top X\right)\right) \end{aligned} \tag{C.1}$$

Therefore, the Bayes optimal form is $F(X) = \log\{\sum_{k=1}^{K}\exp(\tilde{\alpha}_k + \gamma_k^\top X)\}$, where $\tilde{\alpha}_k = \log\pi_k - \frac{1}{2}\mu_k^\top\Sigma^{-1}\mu_k + \log\frac{p_1}{p_0}$ and $\gamma_k = (\Sigma^{-1}\mu_k)^\top$ for $k = 1, \cdots, K$. However, this form may be redundant in parameterization. When we assume that $\gamma_1, \gamma_2, \cdots, \gamma_K$ are linearly independent, for any decomposition of X into $(X_{(1)}, \cdots, X_{(K)})$, there exists the non-singular matrix $A \in \mathbb{R}^{p\times p}$ such that $A\gamma_k = (0, \ldots, 0, \beta_k^\top, 0, \cdots, 0)^\top$ for any $k \in \{1, 2, \cdots, K\}$, where $\beta_k \in \mathbb{R}^{|X_{(k)}|}$. For example, we set

$$A = (B(R^\top R)^{-1}R^\top) + C(I_p - R(R^\top R)^{-1}R^\top), \tag{C.2}$$

where

$$B = \begin{pmatrix} \beta_1 & 0_{p_1} & \cdots & 0_{p_1} \\ 0_{p_2} & \beta_2 & \cdots & 0_{p_2} \\ \vdots & \vdots & \ddots & \vdots \\ 0_{p_K} & \cdots & \cdots & \beta_K \end{pmatrix}, \tag{C.3}$$

$R = (\gamma_1, \cdots, \gamma_K)$ and C is a square matrix of size p. Then $AR = B$, or equivalently $A\gamma_k = (0, \cdots, 0, \beta_k^\top, 0, \cdots, 0)^\top$ for any $k \in \{1, 2, \cdots, K\}$. Assuming that we can choose C such that A is non-singular,

$$
\begin{aligned}
\gamma_k^\top X &= (A\gamma_k)^\top (A^\top)^{-1} X \\
&= (0, \cdots, 0, \tilde{\beta}_k^\top, 0, \cdots, 0)(Z_{(1)}, \cdots, Z_{(K)})^\top \\
&= \tilde{\beta}_k^\top Z_{(K)},
\end{aligned}
$$

where $Z_{(k)} = X_{(k)} A^{-1}$. The existence of such a transformation A confirms that the optimal form $F(X) = \log\{\sum_{k=1}^K \exp(\alpha_k + \gamma_k^\top X_{(k)})\}$. Thus, the quasi-linear predictor is naturally derived when we incorporate the heterogeneity as a normal mixture assumption and modify the optimal form appropriately.

Appendix D : The quasi-linear logistic with L_2 regularization

Here we derive equation (10). Consider the quasi-linear logistic model with L_2. Let us begin with the log likelihood of θ. The gradient vector and Hessian matrix are described as

$$
\begin{aligned}
\frac{\partial l(\theta)}{\partial \theta} &= \sum_{i=1}^n (Y_i - \pi_i) \frac{\partial Q_i}{\partial \theta}, \\
\frac{\partial^2 l}{\partial \theta \partial \theta^\top} &= \sum_{i=1}^n \frac{\partial^2 Q_i}{\partial \theta \partial \theta^\top}(Y_i - \pi_i) - \frac{\partial Q_i}{\partial \theta} \frac{\partial \pi_i}{\partial \theta^\top}.
\end{aligned}
$$

The L_2 penalized log-likelihood is defined by

$$
l^{\mathrm{ridge}}(\theta, \lambda) = l(\theta) - \frac{1}{2}\lambda_0 \sum_{k=1}^K \alpha_k^2 - \frac{1}{2} \sum_{k=1}^K \lambda_k \beta_k^\top \beta_k,
$$

hence we get the gradient vector and Hessian matrix as

$$
\begin{aligned}
\frac{\partial l^{\mathrm{ridge}}}{\partial \theta} &= \frac{\partial l}{\partial \theta} - R_1 \\
&= W^\top (Y - \Pi) - R_1, && \text{(D.1)} \\
\frac{\partial^2 l^{\mathrm{ridge}}}{\partial \theta \partial \theta^\top} &= \frac{\partial^2 l}{\partial \theta \partial \theta^\top} - R_2 \\
&= W^\top V W - R_2, && \text{(D.2)}
\end{aligned}
$$

respectively, where $R_1 = (\lambda_0 \alpha_1, \cdots, \lambda_0 \alpha_K, \lambda_1 \beta_1^\top, \cdots, \lambda_K \beta_K^\top)^\top$ and $R_2 = \mathrm{diag}(\lambda_0 I_K, \lambda_1 I_{p_1}, \cdots, \lambda_K I_{p_K})$.

The MLE with ridge regularization of θ is updated by Fisher's scoring method as

$$
\begin{aligned}
\theta^{(t+1)} &= \theta^{(t)} + \left\{ \mathrm{E}\left[-\frac{\partial^2 l^{\mathrm{ridge}}(\theta^{(t)})}{\partial\theta\partial\theta^\top} \right] \right\}^{-1} \frac{\partial l^{\mathrm{ridge}}(\theta^{(t)})}{\partial\theta} \\
&= \theta^{(t)} + \left[\sum_{i=1}^{n} \pi_i^{(t)}(1-\pi_i^{(t)}) \left(\frac{\partial Q_i}{\partial\theta} \frac{\partial Q_i}{\partial\theta^\top} \right)\bigg|_{\theta=\theta(t)} + R_2 \right]^{-1} \left\{ \sum_{i=1}^{n} (Y_i - \pi_i^{(t)}) \frac{\partial Q_i}{\partial\theta}\bigg|_{\theta=\theta^{(t)}} - R_1 \right\} \\
&= \theta^{(t)} + \left(W^{(t)\top} V^{(t)} W^{(t)} + R_2 \right)^{-1} \left\{ W^{(t)\top}(Y - \Pi^{(t)}) - R_1 \right\} && \text{(D.3)} \\
&= \left(W^{(t)\top} V^{(t)} W^{(t)} + R_2 \right)^{-1} W^{(t)\top} V^{(t)} \left\{ W^{(t)\top}\theta^{(t)} + {V^{(t)}}^{-1}(Y - \Pi^{(t)}) \right\} && \text{(D.4)} \\
&= \left(W^{(t)\top} V^{(t)} W^{(t)} + R_2 \right)^{-1} W^{(t)\top} V^{(t)} Z^{(t)}, && \text{(D.5)}
\end{aligned}
$$

where $Z^{(t)} = W^{(t)\top}\theta^{(t)} + {V^{(t)}}^{-1}(Y - \Pi^{(t)})$. We note that we regularize α by λ_0 to avoid the computational difficulty of calculating the inverse matrix, although the intercept parameters should not be regularized. Thus, we derive equation (10).

Appendix E : The derivatives of the quasi-linear score

Here we derive equation (15). Let $\phi = \phi(z)$ be any invertible, twice differentiable and real-valued function. We consider that X_k includes intercept (i.e. $X_{(k)} = [1, X_{(k)1}, \cdots, X_{(k)p_k}]$). Then, the derivative of G by θ_l is

$$
\frac{\partial G}{\partial \theta_\ell} = \frac{\phi(\theta_\ell^\top X_{(\ell)})}{\dfrac{\partial \phi}{\partial z}\left(\phi^{-1}\left(\displaystyle\sum_{k=1}^{K} \phi(\theta_k^\top X_{(k)}) \right) \right)} X_{(\ell)}.
$$

Therefore, $\phi(z) = \exp(z)$, we observe that

$$
\begin{aligned}
\frac{\partial Q}{\partial \theta_\ell} &= \frac{\exp(\theta_\ell^\top X_{(\ell)})}{\sum_{k=1}^{K} \exp(\theta_k^\top X_{(k)})} X_{(\ell)} \\
&= S_\ell X_{(\ell)} && \text{(E.1)}
\end{aligned}
$$

Thus, we derive equation (15).
