# Supplementary material for: Quasi-linear score for capturing heterogeneous structure in biomarkers
Source: BMC Bioinformatics. 2017 Jun 19;18:308. doi: 10.1186/s12859-017-1721-x (PMC5477283; doi:10.1186/s12859-017-1721-x)
Supplement: Supplementary file 2 — R source code of the parameter estimation of the quasi-linear logistic model. In this file, we introduce the R source code of the parameter estimation of the quasi-linear logistic model, which was used for Simulation and Application. (PDF 12 kb) [file 12859_2017_1721_MOESM2_ESM.pdf]

R source code of the parameter estimation of the quasi-linear logistic model

```
QLL=function(x,y,p.ele,learn.rate=1,reg = 0.01,count.max = 20,step.max = 1000){
  ###getting sizes###
  p = dim(x)[2] ; block = length(p.ele)
  p.ele2 = 0 ; for(k in 1:block){p.ele2 = c(p.ele2,sum(p.ele[1:k]))}

  tmp = x ;  x = list() ; for(k in 1:block){x[[k]] = tmp[,(p.ele2[k]+1):p.ele2[k+1]]}

  ###define some functions###
  f = function(x,alpha,beta){val = exp(alpha + as.matrix(x)%*%beta) ; return(val)}
  F = function(x,alpha,beta){val = log(apply(mapply(f,x,alpha,beta),1,sum)) ; return(val)}
  pi.f = function(x,alpha,beta){val = exp(F(x,alpha,beta));val = val/(1 + val) ; return(val)}

  partf = function(x,y,alpha,beta){
    val = mapply(f,x,alpha,beta)
    pi = pi.f(x,alpha,beta)
    W = val ; for(k in 1:block){W = cbind(W,W[,k]*x[[k]])}
    W = as.matrix(W/apply(W[,1:block],1,sum))
    V = diag(pi*(1-pi))
    score = as.vector((y-pi)%*%W)
    hesse = t(W)%*%V%*%W
    return(list(score,hesse))
  }

  ###calculate###
  theta = list() ; theta[[1]] = rep(0, p + block)
  i = 1 ; count = 0
  while(count <= count.max && i < step.max){
        theta.tmp = theta[[i]]

        alpha.tmp = list()
        for(k in 1:block){alpha.tmp[[k]] = theta.tmp[k]}

        beta.tmp = list()
        for(k in 1:block){
            beta.tmp[[k]] = theta.tmp[(block+p.ele2[k]+1):(block+p.ele2[k+1])]
            }

        tmp = partf(x,y,alpha.tmp,beta.tmp)
        vinv = try(solve(tmp[[2]]),silent=TRUE)
        if(class(vinv)=="try-error"){vinv = ginv(tmp[[2]])}
        theta.tmp = as.vector(theta[[i]] + learn.rate*(vinv%*%tmp[[1]]))
        theta[[i+1]] = theta.tmp
        if( sum( abs(theta[[i+1]]-theta[[i]]) ) > (length(theta[[1]])*reg) ){
        count = 0
        }else{
        count = count+1
        }
        i = i+1
    }

  ###store###
  alpha.tmp = list()
  for(k in 1:block){alpha.tmp[[k]] = theta[[i]][k]}

  beta.tmp = list()
  for(k in 1:block){
  beta.tmp[[k]] = theta[[i]][(block + p.ele2[k] + 1):(block + p.ele2[k+1])]
  }

  coef.copula = c(alpha.tmp,beta.tmp)

  prescore = pi.f(x,alpha.tmp,beta.tmp) ; prescore = log(prescore/(1-prescore))

  trainAUC = ROC(prescore,y)$AUC


  return(list(trainscore=prescore,trainAUC=trainAUC,coef.copula=coef.copula))
  }
```
